# Observation of Human Retinal Remodeling in Octogenarians with a Resveratrol Based Nutritional Supplement

**DOI:** 10.3390/nu5061989

**Published:** 2013-06-04

**Authors:** Stuart Richer, William Stiles, Lawrence Ulanski, Donn Carroll, Carla Podella

**Affiliations:** Eye Clinic 112e, Captain James Lovell Federal Health Care Center, 3001 Green Bay Rd, North Chicago, IL 60064, USA; E-Mails: ilovemabel@aol.com (W.S.); larry.ulanski@gmail.com (L.U.); donn.carroll@gmail.com (D.C.); cjpstella63@gmail.com (C.P.)

**Keywords:** macular degeneration, gene expression, epigenetics, RPE (retinal pigment epithelium) function, VEGF, pharmacogenomics

## Abstract

*Purpose*: Rare spontaneous remissions from age-related macular degeneration (AMD) suggest the human retina has large regenerative capacity, even in advanced age. We present examples of robust improvement of retinal structure and function using an OTC oral resveratrol (RV) based nutritional supplement called Longevinex^®^ or L/RV (*circa* 2004, Resveratrol Partners, LLC, Las Vegas, NV, USA). RV, a polyphenolic phytoalexin caloric-restriction mimic, induces hormesis at low doses with widespread beneficial effects on systemic health. RV alone inhibits neovascularization in the murine retina. Thus far, published evidence includes L/RV mitigation of experimentally induced murine cardiovascular reperfusion injury, amelioration of human atherosclerosis serum biomarkers in a human Japanese randomized placebo controlled trial, modulation of micro RNA 20b and 539 that control hypoxia-inducing-factor (HIF-1) and vascular endothelial growth factor (VEGF) genes in the murine heart (RV inhibited micro RNA20b 189-fold, L/RV 1366-fold). Little is known about the effects of L/RV on human ocular pathology. *Methods*: Absent FDA IRB approval, but with permission from our Chief of Staff and medical center IRB, L/RV is reserved for AMD patients, on a case-by-case compassionate care basis. Patients include those who progress on AREDS II type supplements, refuse intra-vitreal anti-VEGF injections or fail to respond to Lucentis^®^, Avastin^®^ or Eylea^®^. Patients are clinically followed traditionally as well as with multi-spectral retinal imaging, visual acuity, contrast sensitivity, cone glare recovery and macular visual fields. Three cases are presented. *Results*: Observed dramatic short-term anti-VEGF type effect including anatomic restoration of retinal structure with a suggestion of improvement in choroidal blood flow by near IR multispectral imaging. The visual function improvement mirrors the effect seen anatomically. The effect is bilateral with the added benefit of better RPE function. Effects have lasted for one year or longer when taken daily, at which point one patient required initiation of anti-VEGF agents. Unanticipated systemic benefits were observed. *Conclusions*: Preliminary observations support previous publications in animals and humans. Restoration of structure and visual function in octogenarians with daily oral consumption of L/RV is documented. Applications include failure on AREDS II supplements, refusing or failing conventional anti-VEGF therapy, adjunct therapy to improve RPE function, and compassionate use in medically underserved or economically depressed third-world countries.

## 1. Introduction

These notable cases support our original publication as well as work presented in India and subsequently published [1,2]. Resveratrol (RV) was discovered in the US in the 1940s, and later found to be an anti-hyperlipidemic medicinal component of grapes and red wine, also extracted from dried roots of the common weed *Polygonum cuspidatum.* In the modern era, RV has been characterized as a promiscuous, gene regulating, small molecular weight, polyphenol phytoalexin demonstrating broad-spectrum cell receptor and microRNA activity. It protects cells against mitochondrial DNA mutations, has “anti-aging” SIRTUIN activation /deactivation properties, modulates inflammation and most recently mitigated the earliest atherosclerotic marker (loss of flow-mediated dilatation) in a Japanese human clinical trial [3]. The second international conference on the biology of RV [4] and two major literature reviews have broad medical interest [5,6]. There are nearly 3500 papers in PubMed concerning some aspect of RV’s ability to inhibit carcinogenesis at all three stages of initiation, promotion, and progression.

The cardio-protective potential of RV, a major factor underlying the so called “*Red Wine French Paradox*” is equally impressive, in that RV itself can directly protect isolated hearts from ischemia reperfusion injury [7]. In fact, RV protects most vital organs including the kidney, heart, and brain from ischemic reperfusion injury. Broad based cardiac protection is conferred by RVs’ diverse roles as intracellular antioxidant, anti-inflammatory modulator (decreasing COX-2, C-reactive protein and TNF), anti-hypertensive, anti-cholesterol, anti-platelet, nitric oxide synthase expression inductor as well as its ability to regulate angiogenesis in the heart and inhibit neovascularization in the rat retina [5,6,7]. A substantial body of evidence strongly supports the notion that RV mediated cardio-protection is achieved by a unique and often overlooked mechanism called preconditioning. This means the heart is protected prior to a myocardial infarction or chronic ischemia, largely via release of nitric oxide, but also heme oxygenase and adenosine.

RVs’ vascular attributes have special clinical significance and promise for age-related macular degeneration (AMD), due to the debilitating eye diseases’ association with cardiovascular disease risk factors and aging. The emerging importance of sub-retinal choroidal blood flow is now thought to play a pivotal role in AMD. By providing vascular enhancement, small molecular weight cytoprotective nutritionals provide additional vascular enhancement beyond other nutritional factors, *i.e.*, multivitamins, the carotenoids lutein and zeaxanthin. The latter have been studied by the US National Eye Institute, Age Related Eye Disease Study (AREDS) and within our clinic via three placebo controlled, double masked randomized controlled clinical studies involving multivitamins, lutein and most recently zeaxanthin, published during the last two decades [2,8,9].

## 2. Method

*Selecting an oral OTC-RV AMD supplement*: Given there are 432 brands of RV supplements listed at the *Natural Medicines Comprehensive Database*, careful selection of a non-prescription “*over the counter*” RV supplement was limited to brands that have undergone dosage or toxicity testing [10].

Likewise, there is concern over bioavailability and stability of RV as it can be photo-isomerized from *trans* to *cis* RV by exposure to ultraviolet radiation. Selection criteria included evaluating efforts taken to preserve and optimize the biological action of RV to come as close as possible to research-grade RV (frozen, sealed vial) or an alcohol extract of grapes (unfiltered red wine) sealed in a dark bottle. It has been experimentally determined in animals that RV based products provided with additional small natural molecules work synergistically rather than additively and therefore stand the best chance of working in severe and progressive chronic disease. Molecular synergism is believed to be the reason why red wine exerts such a profound biological effect at a relatively low dose of polyphenols—three to five glasses of red wine providing only 180–300 mg of total polyphenols. 

A product called Longevinex^®^ (Resveratrol Partners, LLC, Las Vegas, NV, USA)-hereafter L/RV, was selected that provides 100 mg micronized/microencapsulated *trans*-RV. Micronization dramatically increases the blood concentration of RV. Enfolded in plant starches and dextrins, microencapsulated supplements uniquely protect their components from light, heat and oxygen. The L/RV formulation includes a proprietary blend of other red wine polyphenols, namely quercetin and ferulic acid along with 1200 mg vitamin D3, and a copper/iron/calcium binding molecule called IP6 (inositol hexaphosphate). L/RV has demonstrated synergism at two academic centers and the National Institute of Health [11,12]. Studies show quercetin enhances the immediate bioavailability of RV. Vitamin D3 is important for its ability to exert control over a broad number of genes including those involved in innate immunity, inflammation and vascular calcification. RV increases sensitivity of the vitamin D receptor. Serum 25(OH)D3 liver reserve status is inversely associated with early AMD in the Third National Health and Nutrition Examination Survey, though doubts over that association have recently been expressed.

In animal models, L/RV demonstrated broad superior cardiovascular disease advantage by greater reduction of cardiac fibrosis (scar area), better coronary artery blood flow, far better aortic blood flow and left-ventricular pumping power (left ventricular pressure) compared to RV (or aspirin). In fact, L**/**RV exceeds the effect of RV in 15 of the 25 top microRNA’s implicated in an excised rodent heart model of heart attack and largely restored the pre-event microRNA pattern [11]. MicroRNAs are short non-coding RNAs that mesh with messenger RNA to silence certain genes and are now considered the epigenetic “*guiding hand of the human genome*” [13]. With respect to the retina, L/RV was shown under microRNA analysis (rodent heart tissue) to exhibit many-fold greater down-regulation of microRNA that control HIF-1 hypoxia inducing factor and VEGF-vascular endothelial growth factor genes involved in choroidal neovascularization (angiogenesis), compared to RV alone [12]. These are the critical genes involved in exudative AMD.

In the first human RCT, L/RV produced favorable results (improvement of reduced flow-mediated dilatation, the first sign of atherosclerosis, while decreasing serum insulin, insulin resistance and C-reactive protein) without side effects [3].

*Clinical Safety Considerations*: In the event of inadvertent overdose there is concern that RV may turn from antioxidant to a pro-oxidant. RV characteristically exhibits a U or J shaped risk curve, being cardio-protective between 175 and 350 mg human equivalent dose (HED) in rodents, and cytotoxic (cell killing) at 10-times higher dose (between 1750 mg and 3500 mg HED)—3500 mg being universally lethal to rat hearts. Significantly, L/RV (Longevinex^®^) has been shown to exhibit an unparalleled margin of safety at high doses, exhibiting an L-shaped toxicity curve up to 2800 mg HED whereas 1750 mg becomes a potential pro-oxidant and increases scarring in an induced model of rodent heart attack. This is a margin of safety superior to RV alone.

As RV and L/RV bind to copper and iron, these supplements are contraindicated in growing children, pre-menopausal females and patients with anemia. After ruling out anemia, our octogenarian patients were prescribed one capsule daily L/RV and followed clinically. L/RV was compassionately provided when no other options were available beyond the Vision Impairment Service Team (low vision and blind-rehabilitation services).

*Consent/IRB and Safety*: The use of a marketed product as part of medical practice for an individual patient does not require the submission of an IND (Investigational New Drug). However, oversight was requested and approved by the Chief of Staff and IRB (Hines DVA, Chicago, IL, USA). The “medication” used has exhibited good safety and freedom from side effect at the recommended dosage (one capsule per day) among non-anemic subjects over eight years [14]. FDA animal and human toxicity data, unusual for a dietary supplement, has recently been completed (pending publication). Notations were made in the chart regarding the patients’ willingness to take a nutriceutical pill every day and the fact they were out of options according to retinal specialist consultation. Patients secured L/RV on their own, except in the case of inpatients (*i.e.*, Case 2) who secured the product under the auspices of our Chief of Pharmacy. 

*Retinal Structure*: *Retinal Spectral Separation Image* were obtained with an ARIS^®^ automated retinal imaging system 110 (Visual Pathways, Inc., Prescott, AZ, USA) camera. Compared with traditional fundus photographs, we use spectral (visible/IR) separation images for AMD patients, because of their greater sensitivity in identifying intra-retinal pathology (*i.e.*, retinal drusen that increase in size and volume in AMD), the critical underlying blood supply underneath the retina (*i.e.*, the choroidal network that becomes less dense in AMD) and the macular pigment optical density distribution typically diminished in AMD patients.

Traditional colored fundus photographs were also derived through simple wavelength recombination. *Retinal Pigment Epithelium (RPE) auto fluorescent images* were obtained with the Canon CX1^®^ clinical fundus camera (Canon Medical, Canon USA, Inc., Melville, NY, USA) employing 555 ± 25 nm excitation/640 nm barrier filters. Excessive accumulation of lipofuscin granules in the lysosomal compartment of RPE cells represents a common downstream pathogenetic pathway in various hereditary and complex retinal diseases including AMD [13]. Compared with fluorescein angiography, *in vivo Retinal Spectral Domain Optical Coherence Tomographic (SD OCT) images* were obtained with the RTVue^®^ instrument (OptoVue, Freemont, CA, USA). OCT provides precisely aligned high-resolution *in vivo* histologic sagittal retinal section and thickness images. The OCT highlights retinal alterations in morphology, structure and reflectivity, facilitating baseline and serial clinical evaluation of the retinal layers.

*Visual Function* well known to be impaired in AMD, was measured with several clinical instruments. *Clinical best-refracted Snellen acuity* was taken in a semi-darkened room using a digital projection system (M & S Technologies, Skokie, IL, USA). The *contrast sensitivity function (CSF)*, a measure of how an eye sees large objects (low spatial frequencies at 1.5 and 3 cycles/degree) and small objects such as Snellen letters (higher spatial frequencies, *i.e.*, 18 cycles/degree)—*x* axis, at differing contrasts—*y* axis. The area under the curve of the resulting CSF at five spatial frequencies was measured with The Functional Vision Analyzer^®^ (Stereo Optical, Chicago, IL, USA) with best refraction. *Photo-stress cone glare recovery in seconds* to a bright flash, a measure AMD induced retinal-RPE dysfunction, was measured with a clinical Macular Disease Detection MDD-2^® ^device. (Health Research Science, LLC, Lighthouse Pt, FL, USA). 

## 3. Results

**Figure 1:**
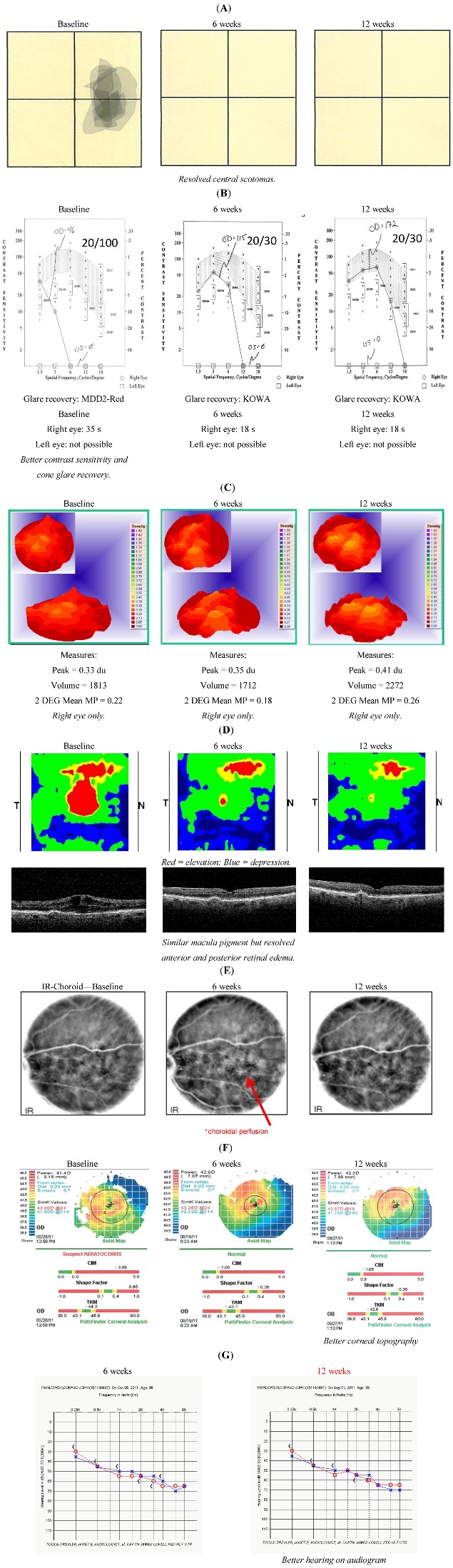
*Case 1*. An 86 y/o morbidly obese male with multiple co morbidities and advanced AMD who is able to read again by three weeks, and whose Snellen visual acuity improves by seven lines at the six weeks visit. Interestingly, in addition to better IR choroidal circulatory images, his bilateral topographic corneal distortions and hearing exam both improve during the same time period.

**Figure 2:**
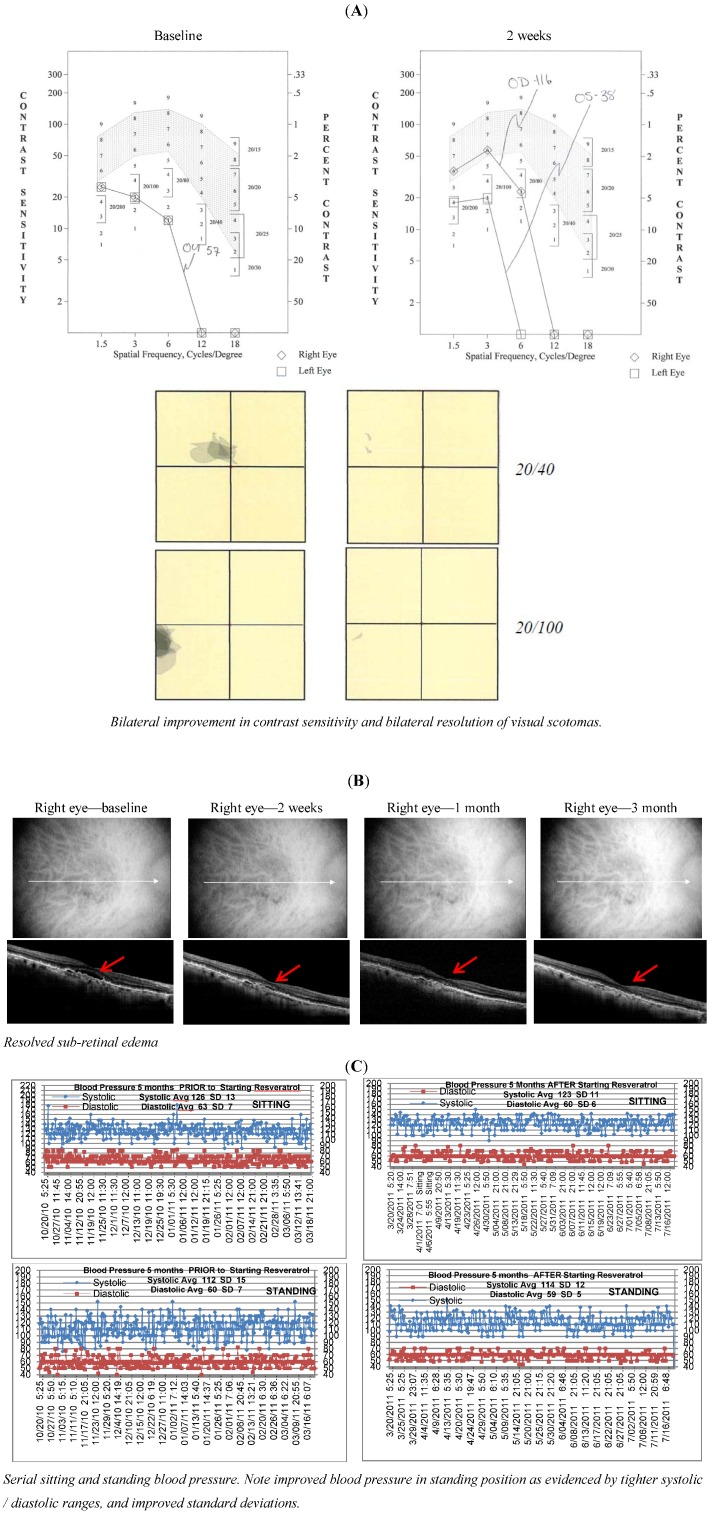
*Case 2*. An 88 y/o female with bilateral wet AMD, who cannot read or identify faces. She adamantly refused intravitreal anti-VEGF treatments, after appeals by two ophthalmologists. Four days after beginning RV/L, she reports a hole opening up in the vision in her better eye, allowing her to see faces. Subsequent measurement at two weeks shows bilateral improvement in visual function and near resolution of retinal fluid. Interestingly, her unremitting hypotension and syncope have not been symptomatic, nor has she experienced chronic migraines since she started L/RV some five months ago.

**Figure 3:**
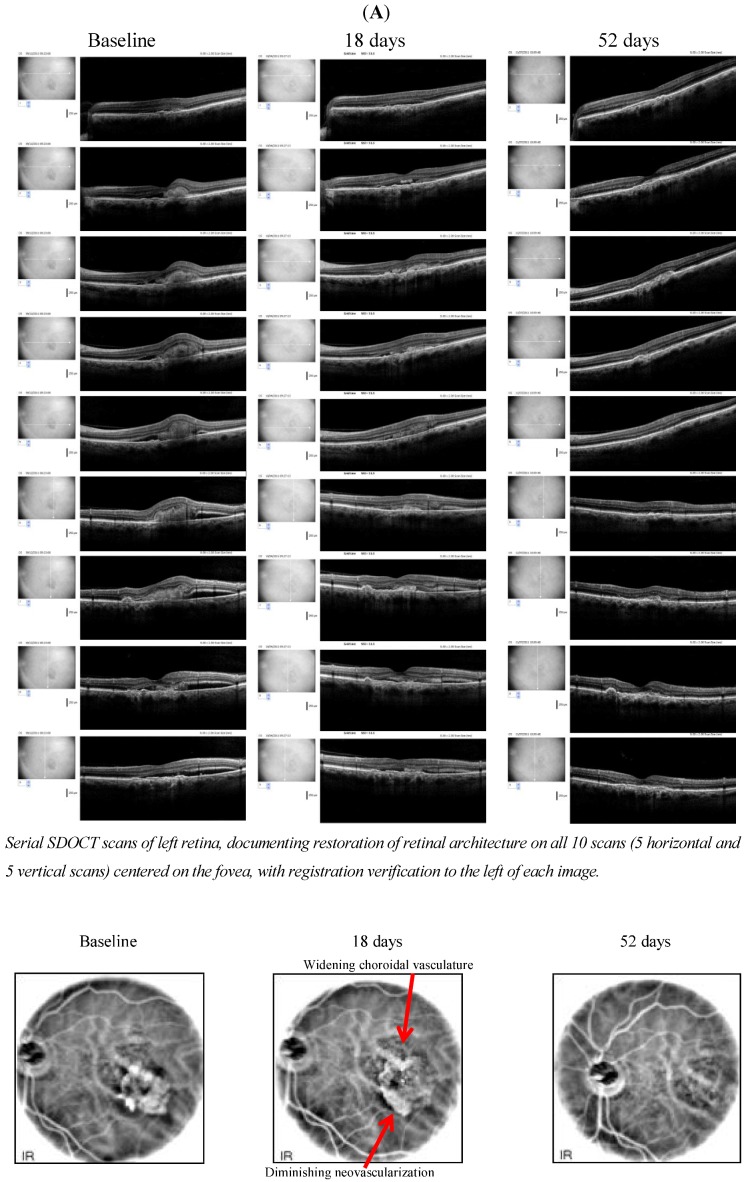
*Case 3*. A younger 75 y/o Vietnam Veteran w prolonged post-traumatic stress disorder, diabetes and dry AMD × 10 years who developed wet AMD in his left eye six months ago but adamantly refused repeated requests to do invasive diagnostic (fluorescein angiography) and intravitrealLucentis^®^ injections or any other type of injection(s). He started L/RV and reported better vision in five days and passed his driver’s license after seven capsules. At his two week clinical appointment, we see objective retinal and visual restoration, similar to anti-VEGF therapy.

Figure 1, Figure 2, Figure 3 present octogenarian L/RV AMD patients for whom all other therapeutic measures had been exhausted (ARED and AREDS II supplements, anti-VEGF treatments *etc.*), or the patient either refused intra-vitreal injections or were classified as non-candidates by conventional retinal ophthalmologic evaluation. Subjects (two males and one female) were either US WW II veterans or veterans of the Korean Conflict (Case 2) and Vietnam Era (Case 3) receiving eye care at the James A Lovell Federal Health Care Facility, North Chicago, IL, USA.

## 4. Discussion

L/RV, a nutritional supplement, demonstrated wide-ranging ocular, visual and systemic health benefit(s) in octogenarian AMD patients with no medical options other than “*wait and see*”, low-vision care and blind rehabilitation. In AMD, objective retinal imaging revealed higher intra-retinal reflectivity denoting distinct differentiation of individual retinal striatum and retinal microstructure [2]. Specifically, we have observed a brighter IS/OS junction (perhaps mitochondrial renewal) and a brighter and flatter retinal pigment epithelium (presumably more melanized) with less shadowing of underlying structures as visualized by OCT imaging, suggesting both improved anterior retinal and posterior retinal photoreceptor/RPE health. There were rapid Avastin^®^, Lucentis^®^ and EyLea^®^-like anti-VEGF effects. This improvement in structure was accompanied in all cases not only by broadly superior conventional Snellen Visual Acuity, but also greater comprehensive visual function: better contrast sensitivity, scotoma resolution/improvement and quickened photo-stress glare recovery. In all cases, bilateral improvements were observed - albeit with only modest improvements in function in the more severely diseased eye.

Octogenarians report an improvement in physical stamina shortly after starting L/RV with Medical-center documented co-morbidity improvements. We have observed improved audiograms (patient 1, another unpublished). Patient 1 also demonstrated an unanticipated smoother corneal epithelial topographic scan. Patient 2 improved her systemic hypotension and syncope the very reason she has been hospitalized for two years. Collectively, these phenomena suggest a return of structure and function in the “*oldest-old*” toward a more youthful physiological state and support our previous two publications [1,2].

These cases illustrate the potential benefit of using a mineral-chelating matrix to thwart the accumulation of redox-reactive divalent copper and iron, since age-related accumulation of these minerals parallels the onset of age-related retinal disease. Significantly, age-related over mineralization controls genes involved in AMD. 

Hypoxia/ischemia/angiogenesis-related damage, seen in aged and diseased retinal tissues, are controlled by “*master ischemic*” micro RNAs. In this context, RV and more so L/RV in murine heart tissue, were found to exert a profound effect over micro RNAs, restoring expression to pre-event profiles largely via control of the hypoxia-inducing-factor gene (HIF-1: micro RNA-20b) and the vascular endothelial growth factor gene (VEGF: micro RNA-539), though L/RVs epigenetic effects were not limited to these genes. L/RV exhibits a 1.82 times greater differentiation over micro RNA-539 (up +314.6 compared to plain RV, up +172.4) and a 7.22 times greater down-regulation over micro RNA-20b (down −1366.0 compared to plain RV, down −189.0) [11]. Therefore, plain RV may not produce results equivalent to those demonstrated in these subjects, and higher doses are not recommended as this theoretically induces a pro-oxidant effect.

The metabolically active retina is uniquely characterized by daily disc shedding, renewal of photoreceptors, high oxygen/blood perfusion levels and exposure to radiation. This suggests a regenerative capacity likely hindered by local inflammation and oxidation. In some cases we see serous elevation of the retina with altered photoreceptor outer segments and highly reflective outer-plexiform degenerative bodies, thought to represent cone distress and rod engorgement. We have observed these phenomena to often disappear after starting L/RV. Despite the capacity for repair and regeneration, once photoreceptors or inner retinal neurons have degenerated severely, they are not spontaneously replaced in mammals, as seen in the worse functioning eyes in this case series. However, we unexpectedly observed diminished RPE auto fluorescence in several patients taking L/RV, as reported by others, with RV, *in vitro* suggesting innate regenerative capacity. We have had AF images independently verified. 

Is it possible that we are seeing hormesis sustained nascent stem cell regeneration? The retina retains a regenerative/repair capacity for replacement of lost neurons via differentiation of Müller glia cells to a progenitor (stem-cell like) state. Non-telomeric double-strand DNA breaks may exhaust the stem cell pool, impairing cellular repair. Small nutriceutical molecules are essential for double-strand DNA-break repair. Furthermore, RV may exert a surprising ability to facilitate stem cell survival and thus tissue renewal. Stem cells are normally generated at sites of tissue damage. Recent studies suggest certain small antioxidant molecules like RV, via their ability to quell free radicals, enable endogenously produced or injected stem cells to survive rather than die. Obvious restoration of retinal layers as visualized on digital SD-OCT images suggest the observed retinal cell repopulation and tissue regeneration in these octogenarian patients were likely facilitated by stem cell survival. 

In the heart, RV remarkably normalizes vascularization and promotes capillary budding in damaged cardiomyocytes that have a slow-cell turnover rate [12]. In the retina, RV inhibits new blood vessel outcropping in retinal tissues that have a fast cell turnover rate. RV alone and we believe more so RV combined with other potentiating small molecules, activates autophagy, a catabolic renewal process which involves degradation of a cell’s own debris through lysosomal enzymes, facilitating a coordinated genomic response and eventual greater differentiation (gene expression or silencing) of several longevity proteins. The previously demonstrated beneficial low-dose epigenetic effects of RVand the L/RV nutriceutical multi-molecule mix is believed to mimic that observed with studies involving modest-dose red wine that prompts activation of adenosine receptors. Adenosine, a cell-protective/vaso-dilative nucleoside, is known to prevent damage in tissues deprived of oxygen (hypoxia, ischemia), as typically occurs in AMD.

**Figure A1:**
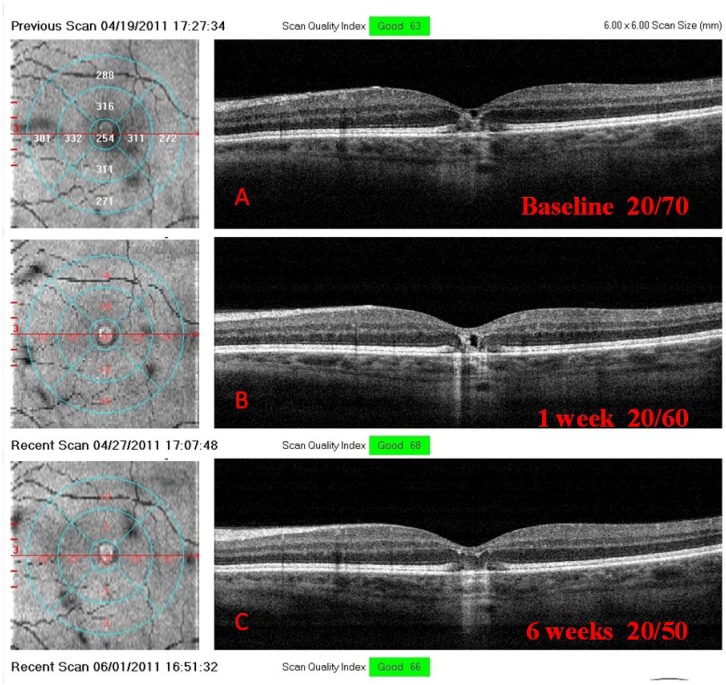
(**A**) SD OCT horizontal left macula retinal scan of a 60 y/o male with Stage 2 Adult Foveo-Macular Dystrophy, a variant of Vitelliform Macular Dystrophy. The L retina is more effected with 20/70 VA and central 4 degree fovealAmsler grid distortion. (The R eye is 20/20 with form fruste findings—not shown). Diagnosis made in 2007 by William KM Shields, Retina & Macula Specialists, Olympia, WA, USA. A sister and both parents similarly affected. (**B**) After 5 days on Longevinex^®^, patient reports darkening and less distortion of the central Amsler grid with 1 line VA improvement to 20/60 with the same eyeglasses. (**C**) At 6 weeks, the Amsler grid shows continual darkening and the patient reports that the central distorted area has moved to the right side of the grid. Vision is now 20/50, 2 lines better than baseline. The SDOCT images show increasing tissue mass and reformation of the fovea, denoting possible *in vivo* stem cell activity. Note images are registered, within the fovea, according to the 840 nm IR ETDRS grid (left) simultaneously imaged on the retina. (Images courtesy of Donn Carroll, Private Practice, WA Medical Vision Center, Morton, WA, USA).

Treatment-resistant AMD patients, even the oldest-old, may not be “beyond hope”. Only treatment of a larger number of cases, within a research protocol, will reveal with what reliability. However, there is reason to believe that the clinical phenomena we observe are curative and not due to placebo effects. We have observed recurrence of visual decline and altered retinal morphology with temporary cessation of L/RV, suggesting cause and effect relationship. Of course, unknown mechanisms and explanations for these phenomena might be at play. Retinal drusen can spontaneously resolve in 15% of AMD patients. However, one of us (DC) has observed diminution of drusen volume in as little as one week after starting L/RV, albeit in younger patients. He also has documented regeneration of glial tissue and improvement in visual function in a case of familial vitelliform (hereditary) retinal dystrophy. Diseased retinas typically worsen. See Appendix Figure A1.

While this report herald hopes for patients with otherwise intractable cases of neovascular or geographic AMD, much needs to be learned. These three cases represent the most demonstrable visual and structural improvement we have seen, all of whom were non-candidates for anti-VEGF therapy. The oldest patient treated is age 92. L/RV appears to stabilize AMD disease progression and has produced modest improvements in multiple clinical aspects of vision (acuity, contrast, fields, glare recovery). There are no reported side effects.

It is our hope that a nutritional supplement modulating inflammation, inducing micro RNA 20b and 539, mitochondrial renewal and redox reactive mineral sequestration be added to the ophthalmologist’s toolbox. It appears some of the enigmatic biological mechanisms responsible for cases of spontaneous remission of retinal disease are of environment—gene interactive epigenetic origin. Compassionate use, pilot studies and fast track FDA Phase 2 randomized clinical trial of L/RV, all appear warranted.

Anti-VEGF therapy is the prevailing treatment for the fast-progressive “*wet*” form of AMD. Elevated vascular endothelial growth factor (VEGF) appears to be a local problem in the retina rather than a systemic issue. While injected monoclonal antibodies narrowly block VEGF, molecular medicine exerts broader epigenetic control over neovascularization, inflammation, RPE health, blood-flow and possibly tissue regeneration. Because of the broad genomic action of RV and more so L/RV, the health benefits of these small molecules cannot be confined to any particular retinal layer. In these cases notable improvement in vision, but curiously also hearing, blood pressure and vigor were observed among octogenarians. While broad biological action by RV has been exhibited in the animal lab, this may be the first documented presentation of positive tissue-wide and system-wide biological effects in humans. 

We have observed speedy anatomic and bilateral visual benefit with ingestion of a red wine extract combined with vitamin D and a redox-active divalent metal sequestering agent. In AMD, we conjecture that L/RV may reduce the number of required injections, benefit some patients that fail anti-VEGF therapy, as well be useful to patients who are averse to needle infusion or cannot afford standard therapy. Side effects were not reported.

## 5. Conclusions

In our ongoing clinical experience, treatment-resistant macular degeneration patients are not beyond hope, unless scarring or absolute wide spread retinal/choroidal histopathologic tissue destruction has occurred. Furthermore, there is reason to believe the beneficial nutrient—induced retinal structural and functional visual effects in these case presentations, are not due to a placebo effect. Nonetheless, randomized placebo controlled molecular medicine clinical studies, to confirm the proposed beneficial effects of low dose epigenetic nutriceutical intervention beyond AREDS 2 supplementation, are warranted.

## Declaration

Presented at the Association for Research in Vision and Ophthalmology Meeting (ARVO), Fort Lauderdale, FL, USA, poster #286, 6 May 2012.
